# Supplementary material for: GPS Pipeline: portable, scalable genomic pipeline for Streptococcus pneumoniae surveillance from Global Pneumococcal Sequencing Project
Source: Nat Commun. 2025 Sep 24;16:8345. doi: 10.1038/s41467-025-64018-5 (PMC12460886; doi:10.1038/s41467-025-64018-5)
Supplement: Supplementary file 1 — Supplementary Information [file 41467_2025_64018_MOESM1_ESM.pdf]

Supplementary Table 1. **Details of quality control parameters of the pipeline**

Type of Quality Control	Quality Control Parameter	Default Value
Read	Minimum base count<br>(not directly accessible, based on the multiplication of minimum assembly length and minimum sequencing depth)	≥38,000,000 bp
Assembly	Maximum contig count	≤500 contigs
	Minimum assembly length	≥1,900,000 bp
	Maximum assembly length	≤2,300,000 bp
	Minimum sequencing depth	≥20x
Taxonomy	Minimum *S. pneumoniae* percentage in reads	≥60%
	Maximum non-*Streptococcus* genus percentage in reads	≤2%
Mapping	Minimum reference coverage percentage by the reads	≥60%
	Maximum non-cluster heterozygous SNP (het-SNP) site count	≤220 het-SNP sites

Supplementary Table 2. **System requirements comparison between Kraken 2 and GTDB-Tk**

	Disk Space	Memory
Kraken 2	8GB<br>(Minikraken v1 database)	8GB<br>(Same size as the database)
GTDB-tk	~106GB<br>(GTDB)	~90GB<br>(for Bacteria)

Supplementary Table 3. **Details of Docker images used in the pipeline**

Docker Image Reference with Tag	Developer	Provided Tool(s)
amancevice/pandas:2.0.2	Alexander Mancevice	Python 3.11.3; pandas 2.0.2
sangerbentleygroup/seroba:2.0.4	Bentley Group	SeroBA v2.0.4; SeroBA database v2.0.4
sangerbentleygroup/spn-pbp-amr:23.10.2	Bentley Group	CDC PBP AMR Predictor release 23.10.2
staphb/ariba:2.14.6	StaPH-B	ARIBA v2.14.6
staphb/bcftools:1.16	StaPH-B	BCFtools v1.16
staphb/bwa:0.7.17	StaPH-B	BWA v0.7.17
staphb/fastp:0.23.4	StaPH-B	fastp v0.23.4
staphb/kraken2:2.1.2-no-db	StaPH-B	Kraken 2 v2.1.2
staphb/mlst:2.23.0-2024-07-01	StaPH-B	mlst v2.23.0; PubMLST database accessed on 1st July 2024
staphb/poppunk:2.6.3	StaPH-B	PopPUNK v2.6.3
staphb/quast:5.0.2	StaPH-B	QUAST v5.0.2
staphb/samtools:1.16	StaPH-B	SAMTools v1.16
staphb/shovill:1.1.0-2022Dec	StaPH-B	Shovill v1.1.0
staphb/unicycler:0.5.0	StaPH-B	Unicycler v0.5.0
wbitt/network-multitool:69aa4d5	WBITT	BusyBox 1.34.1 with awk, bc, grep, gzip, paste, sed, sort, tar, zcat; GNU Bash 5.1.8; GNU Wget 1.21.2; jq 1.6

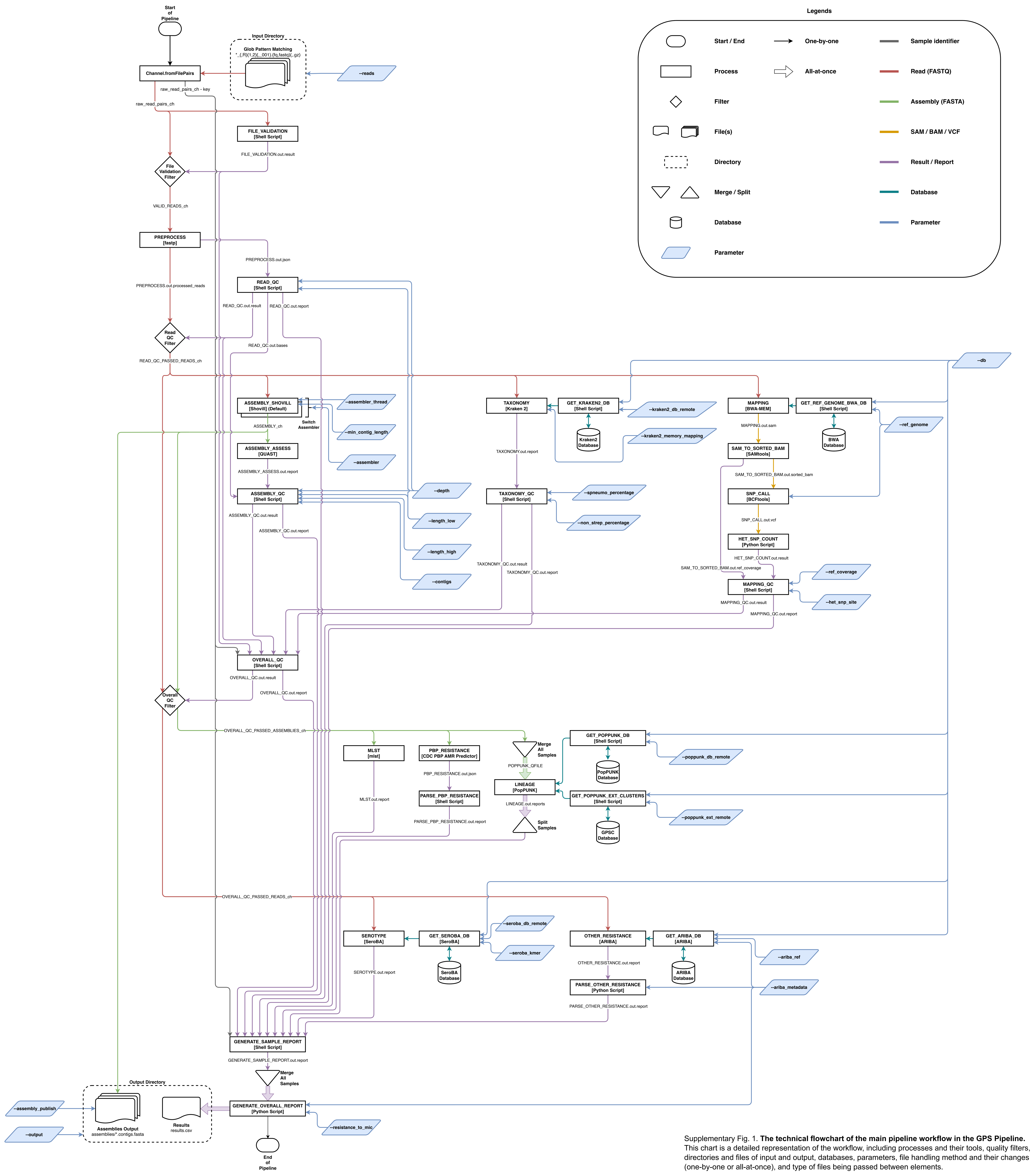

Supplementary Fig. 1. **The technical flowchart of the main pipeline workflow in the GPS Pipeline.** This chart is a detailed representation of the workflow, including processes and their tools, quality filters, directories and files of input and output, databases, parameters, file handling method and their changes (one-by-one or all-at-once), and type of files being passed between elements.

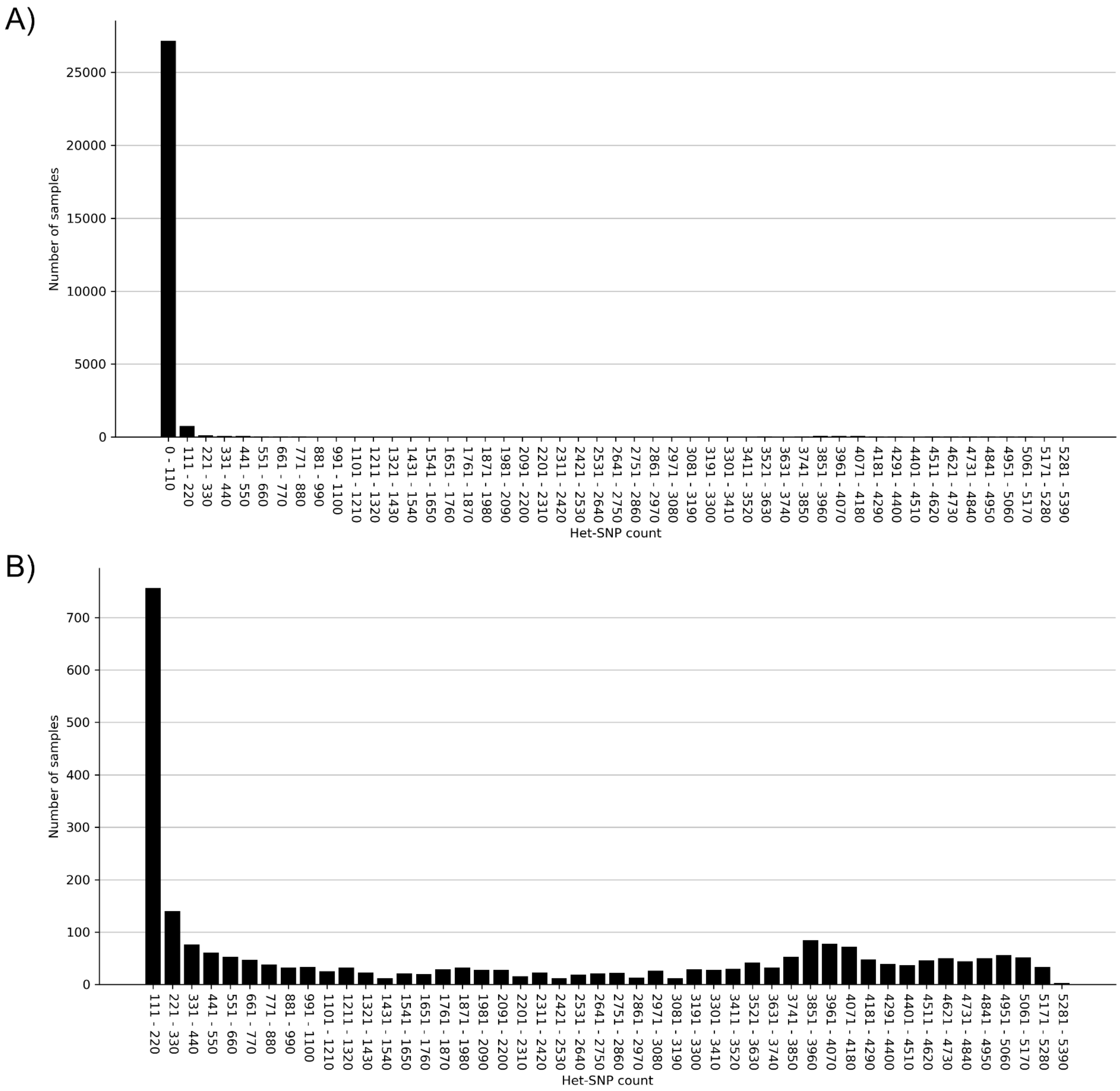

Supplementary Fig. 2. **The Het-SNP counts across 29,913 pneumococcal genomes in the GPS project.** A) Distribution of het-SNP counts across genomes with a bin size of 110. B) The distribution of het-SNP counts when the first bin is excluded. The majority of the genomes fall into the first two bins, with a significant fall-off between the second and third bins. Therefore, we consider a count of het-SNP >220 as insufficient isolate purity.
