# Supplementary material for: GPS Pipeline: portable, scalable genomic pipeline for Streptococcus pneumoniae surveillance from Global Pneumococcal Sequencing Project
Source: Nat Commun. 2025 Sep 24;16:8345. doi: 10.1038/s41467-025-64018-5 (PMC12460886; doi:10.1038/s41467-025-64018-5)
Supplement: Supplementary file 2 — Description of Additional Supplementary Files [file 41467_2025_64018_MOESM2_ESM.pdf]

Description of Additional Supplementary Files

File Name: Supplementary Data 1
Description: Metadata of Poland genomes demonstration dataset

File Name: Supplementary Data 2
Description: Output of the GPS Pipeline on the demonstration dataset

File Name: Supplementary Data 3
Description: List of accession numbers of all published data from GPS Database used in validation
